# Spinal Arteriovenous Fistula, A Manifestation of Hereditary Hemorrhagic Telangiectasia: A Case Report

**DOI:** 10.5811/cpcem.2020.5.47086

**Published:** 2020-07-03

**Authors:** Jodi Spangler, Bjorn Watsjold, Jonathan S. Ilgen

**Affiliations:** University of Washington School of Medicine, Department of Emergency Medicine, Seattle, Washington

**Keywords:** Hereditary hemorrhagic telangiectasia, arteriovenous fistula

## Abstract

**Introduction:**

Hereditary hemorrhagic telangiectasia (HHT) is an autosomal dominant disorder characterized by arteriovenous malformations (AVM). HHT can have neurological manifestations.

**Case Report:**

A 32-year-old woman with a history of HHT presented to the emergency department with acute partial paralysis of the right leg, urinary retention, and right-sided back and hip pain. Magnetic resonance imaging of the spine demonstrated multiple, dilated blood vessels along the cervical spine, diffuse AVMs in the lumbar and thoracic spine, and a new arteriovenous fistula at the twelfth thoracic (T12) vertebral level. Her symptoms improved after endovascular embolization of the fistula.

**Conclusion:**

Spinal AVMs are thought to be more prevalent in patients with HHT. Given the high morbidity of arteriovenous fistulas, early recognition and intervention are critical.

## INTRODUCTION

Hereditary hemorrhagic telangiectasia (HHT) is an autosomal dominant disorder occurring in approximately 1 in 10,000 people, characterized by arteriovenous malformations (AVM) in the gastrointestinal tract, central nervous system, and lungs, mucocutaneous telangiectasia, and recurrent epistaxis.^1^,^2^,^3^ HHT can have neurological manifestations, and, although rare, spinal AVMs are thought to be more prevalent in these patients.^4^ Arteriovenous fistulas (AVF) are direct communications between arteries and veins without a vascular nidus and have a high morbidity if untreated.^5^,^6^

## CASE REPORT

A 32-year-old woman with a history of HHT presented to the emergency department (ED) with acute right hip and flank pain with associated weakness in her right leg.

The patient, as a result of her HHT, had a history of persistent epistaxis requiring embolization and two prior spontaneous subdural hemorrhages that required decompressive craniotomies five years prior to her visit. At that time, computed tomography (CT) and magnetic resonance imaging (MRI) imaging of her spine demonstrated diffusely dilated vessels that extended from her cervical spine to the base of her thoracic spine, causing compression and deformity of the spinal cord. These were suspected to be secondary to dural AVFs and an AVM at the seventh thoracic (T7) vertebral level. Neurosurgical consultants recommended surgical intervention to prevent myelopathy, but she elected not to undergo surgery because of the potential complications of the procedures.

She was otherwise healthy, did not smoke, drink alcohol, or take recreational drugs. She had emigrated from Ethiopia, and her father had had recurrent episodes of epistaxis that were thought to be secondary to HHT, although this was never formally diagnosed.

Two weeks prior to presentation, the patient had sought care in the ED for right hip and flank pain. At that time, her physical examination showed no definite motor weakness, and she was discharged to follow-up with her physician. She had two subsequent ED visits at other hospitals and was documented to have increasing weakness of hip flexion (4/5), knee extension (4/5), and ankle dorsiflexion (3/5). She eventually needed crutches for mobility. Across these three visits, the patient had negative radiographs of the femur, normal CT imaging of the abdomen and pelvis, and normal MRI of the pelvis.

Her pain and weakness progressed until she lost sensation in her right leg, was unable to bear weight secondary to weakness, and was subsequently bedridden. Additionally, she developed increased urinary frequency and difficulty fully evacuating her bladder. Upon her return to the ED, she was noted to have profound (0/5) weakness of right hip flexion, knee flexion, and ankle plantar- and dorsiflexion, as well as significant (2/5) weakness of right knee extension. She had no sensation to pinprick throughout her right thigh, leg, and foot, and had hyporeflexia throughout her right lower extremity. Her plantar reflex in both feet was normal, as were her mental status and cranial nerves. She had intact rectal tone and perineal sensation.

**Image 1:**
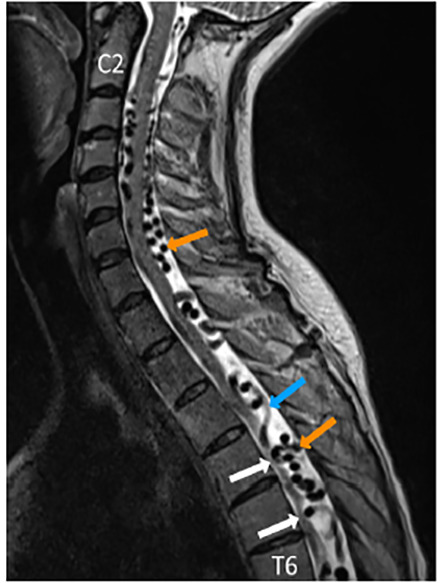
Sagittal magnetic resonance imaging T2-weighted image of the cervical and thoracic spine. Flow voids appear black against white cerebrospinal fluid, and indicate extensive dilated vessels suggestive of dural arteriovenous fistulas. Orange arrows identify vessels perpendicular to the plane of the image, blue arrow identifies vessel at an angle to the plane with relatively increased signal. The white arrows highlight areas of spinal cord being compressed or deformed out of plane by dilated vessels.

**Image 2:**
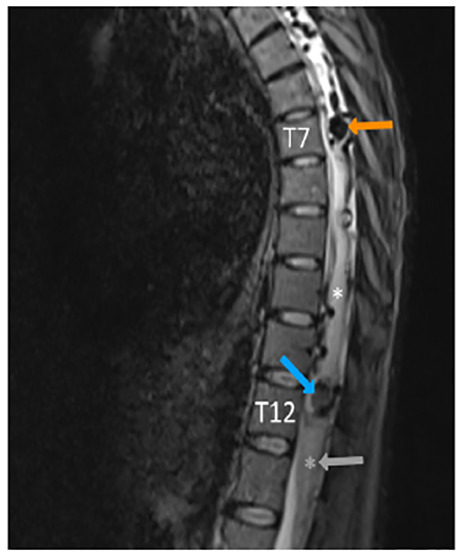
Sagittal magnetic resonance imaging T2-weighted image of the thoracic and lumbar spine. The orange arrow points to a flow void suggestive of an arteriovenous malformation with dilated varix at seventh thoracic (T7). There is a similar dilated varix at T11 to the twelfth thoracic (T12), and the blue arrow shows an area of darker gray suggesting hemosiderin from subacute or chronic hemorrhage. The spinal cord in this image is hyperintense due to venous congestion and edema, possibly worsened by ischemia. For comparison of relative signal intensity, the gray asterisk and arrow at the conus show the same signal level as the cord at the tenth thoracic level surrounding the white asterisk.

Based on her presentation, her providers were concerned for spinal cord compression, dural compression syndrome, and spinal cord ischemia. Neurosurgery was consulted, and MRI of her entire spine was performed. MRI of her cervical, thoracic, and lumbar spine demonstrated diffuse, dilated vessels extending from her brainstem to T12, consistent with her known dural AVF (Image 1). In addition to multiple arterial feeder vessels contributing to the AVF, she had two areas of AVM at T7 and T11-12, and an area of subacute/chronic hemorrhage at T11-12 (Image 2). There was also diffuse abnormality of the spinal cord signal extending from T6 to the conus medullaris that was concerning for multiple processes including edema and ischemia.

**CPC-EM Capsule**

What do we already know about this clinical entity?

Hereditary Hemorrhagic Telangiectasia (HHT) is an autosomal dominant disorder characterized by arteriovenous malformations (AVMs).

What makes this presentation of disease reportable?

A rare case of spinal arteriovenous malformations and fistula in a patient with HHT who presented with neurological symptoms.

What is the major learning point?

HHT can have neurological manifestations, and, although rare, spinal AVMs are thought to be more prevalent in these patients.

How might this improve emergency medicine practice?

Given the high morbidity of arteriovenous fistulas, early recognition and intervention are critical.

The patient underwent angiography and embolization of a feeder vessel to the dural AVF originating at T12 that connected to the AVM at T7, but this procedure incompletely treated the abnormal flow. After the procedure, she discussed further intervention with the neurosurgical service, and again declined surgical intervention. The patient spent four weeks in the hospital postoperatively and in rehabilitation, ultimately regaining normal bladder function and motor function in her right leg.

Two years after this visit, the patient developed left-sided radicular leg pain without loss of motor function. She continued to follow up with neurosurgery but postponed management of the residual AVM due to other complications of HHT, including anemia, recurrent epistaxis, and cardiomyopathy due to high-output heart failure. She developed progressive loss of function in the right leg, and eventually underwent successful embolization of the remaining fistula pouch nearly four years after the initial visit.

## DISCUSSION

HHT is an autosomal dominant disorder characterized by AVMs in the gastrointestinal tract, central nervous system, and lungs, mucocutaneous telangiectasia, and recurrent epistaxis.^1^,^2^,^3^ Thought to be caused by changes in angiogenesis, HHT manifestations develop with increasing age and range from being asymptomatic to life-threatening.^8^ Spontaneous recurrent epistaxis from telangiectasia of the nasal mucosa is the most common symptom and usually the earliest sign of the disease.^1^

This patient’s initial evaluations focused on hip, leg, and flank pain, with multiple unrevealing imaging studies. When she returned with muscle weakness, sensory deficits, and urinary retention, there was concern for acute pathology affecting the spinal cord or peripheral nerves. In an otherwise healthy patient, acute neurologic deficits such as these may have prompted consideration of cauda equina syndrome, inflammatory and autoimmune pathologies including acute transverse myelitis, multiple sclerosis, and Guillain-Barré syndrome, and infectious causes including epidural abscess. In a patient with HHT, one must take into consideration that their vascular abnormalities increase the risk for central and peripheral neurologic insults. Pulmonary AVMs can increase risk of embolic stroke, and cerebral and/or spinal AVMs can cause local ischemia and hemorrhage. Imaging and management should, therefore, correlate physical exam findings with the likely level of the injury.^7^

HHT has neurological involvement in up to 20% of patients, including cerebral and spinal AVMs.^9^ Cerebral AVMs can result in hemorrhage, such as in this patient’s prior spontaneous subdural hematomas. Spinal AVMs are thought to be more prevalent in patients with HHT, although these lesions are rare (estimated prevalence of less than 1% in patients with HHT) with the majority being perimedullary fistulae with complex high-flow angioarchitecture.^3^,^4^ Spinal vascular malformations are categorized as non-shunting lesions (aneurysms) and shunting lesions (AVM and fistulas).^10^ AVFs are a direct communication between arteries and veins without a vascular nidus, and spinal AVF in particular is an abnormal connection between an arterial feeder and a draining vein in the spinal cord dura or arachnoid.^5^,^6^

Perimedullary AVFs commonly develop at the ventral portion of the spinal cord, while dural AVFs commonly develop at the dorsolateral portion of the dura mater.^11^ Dorsal AVFs are typically low flow, and lead to congestive myelopathy that affects the caudal end of the cord regardless of the level of fistula.^12^ The majority of AVFs are idiopathic and detected only when symptoms arise, with acute or subacute presentation, presenting as severe neurological deficits, progressive myelopathy, or subarachnoid hemorrhage.^4^ The most common presenting symptoms, such as in this case, are acute motor deficits.^4^

The diagnosis of spinal AVMs is often delayed and challenging, and significant morbidity can occur before surgical or endovascular intervention.^8^ Lesions detected early are reversible, and the preferred treatment modality for spinal vascular malformations in HHT is endovascular embolization, which also helps to decrease the risk of developing future vascular collaterals.^13^,^14^ If endovascular intervention is unsuccessful, surgery is also an option to interrupt the shunt.^13^ In general, early treatment has a high rate of clinical and angiographic improvement and complications are uncommon.^13^,^15^

## CONCLUSION

Although rare, vascular etiologies such as spinal arteriovenous fistula should be in the differential diagnosis for a patient with HHT presenting with neurological symptoms. Spinal AVF can cause significant morbidity, and given the effectiveness of embolization or surgical intervention, early recognition and treatment are critical.
